# Integrated radiomics, dose-volume histogram criteria and clinical features for early prediction of saliva amount reduction after radiotherapy in nasopharyngeal cancer patients

**DOI:** 10.1007/s12672-022-00606-x

**Published:** 2022-12-30

**Authors:** Lang Zhou, Wanjia Zheng, Sijuan Huang, Xin Yang

**Affiliations:** 1 State Key Laboratory of Oncology in South China; Collaborative Innovation Center for Cancer Medicine; Guangdong Key Laboratory of Nasopharyngeal Carcinoma Diagnosis and Therapy, Sun Yat-Sen University Cancer Center, Guangzhou, 510060 Guangdong Province China; 2 Department of Biomedical Engineering, South China University of Technology, Guangzhou, 510640 Guangdong Province China; 3 Department of Radiation Oncology, Southern Theater Air Force Hospital of the People’s Liberation Army, Guangzhou, 510050 Guangdong Province China

**Keywords:** Acute xerostomia, Saliva amount prediction, Machine learning, Feature importance

## Abstract

**Purpose:**

Previously, the evaluation of xerostomia depended on subjective grading systems, rather than the accurate saliva amount reduction. Our aim was to quantify acute xerostomia with reduced saliva amount, and apply radiomics, dose-volume histogram (DVH) criteria and clinical features to predict saliva amount reduction by machine learning techniques.

**Material and methods:**

Computed tomography (CT) of parotid glands, DVH, and clinical data of 52 patients were collected to extract radiomics, DVH criteria and clinical features, respectively. Firstly, radiomics, DVH criteria and clinical features were divided into 3 groups for feature selection, in order to alleviate the masking effect of the number of features in different groups. Secondly, the top features in the 3 groups composed integrated features, and features selection was performed again for integrated features. In this study, feature selection was used as a combination of eXtreme Gradient Boosting (XGBoost) and SHapley Additive exPlanations (SHAP) to alleviate multicollinearity. Finally, 6 machine learning techniques were used for predicting saliva amount reduction. Meanwhile, top radiomics features were modeled using the same machine learning techniques for comparison.

**Result:**

17 integrated features (10 radiomics, 4 clinical, 3 DVH criteria) were selected to predict saliva amount reduction, with a mean square error (MSE) of 0.6994 and a R^2^ score of 0.9815. Top 17 and 10 selected radiomics features predicted saliva amount reduction, with MSE of 0.7376, 0.7519, and R^2^ score of 0.9805, 0.9801, respectively.

**Conclusion:**

With the same number of features, integrated features (radiomics + DVH criteria + clinical) performed better than radiomics features alone. The important DVH criteria and clinical features mainly included, white blood cells (WBC), parotid_glands_Dmax, Age, parotid_glands_V15, hemoglobin (Hb), BMI and parotid_glands_V45.

## Introduction

Xerostomia is a common side effect of nasopharyngeal carcinoma (NPC) radiotherapy (RT) [1, 2]. It seriously affects the quality of life (QOL) of patients, including oral infection, eating problem, undernutrition, and insomnia [3–5]. Intensity modulated radiation therapy (IMRT) is a technique to better protect the organs at risk (OARs) [6], but parotid glands (PGs) are inevitably included in the irradiation field, causing radiation injury. Accurate prediction could assist early intervention of xerostomia.

It is widely reported that radiomics features improve the performance of xerostomia prediction. Radiomics features of PGs extracted from CT [7–12], CBCT [13], MRI [14–16], and PET [17, 18] reflect the condition of PGs and can be used as an important factor in xerostomia prediction. Van Dijk et al*.* revealed that PGs surface reduction was associated with late xerostomia [8]. Pota et al*.* used radiomics features extracted from CT to predict PGs contraction and explored the relationship between PGs contraction and xerostomia [10]. They found that acute xerostomia was positively associated with PGs contraction, while xerostomia after 2 years of RT is negatively associated with it. Wu et al*.* and Rosen et al*.* both pointed out that the mean Hounsfield units (HU) of PGs was effective for the prediction of xerostomia [13, 19]. It is known that the mean HU of PGs mirrored the density of PGs. Similarly, Belli et al*.* revealed that the gradient of PGs density played an important role in predicting xerostomia after RT [20]. Zhang et al*.* found that the maximum apparent diffusion coefficient of PGs was a sensitive indicator for salivary gland dysfunction, so it was potential to predict xerostomia after RT [21].

Dose-volume histogram (DVH) criteria features were one of the first factors used to predict xerostomia after RT. Many studies showed that the degree of xerostomia after RT decreased as the mean dose of PGs reduced [22–26]. Deasy et al*.* found that PGs function had the least reduction when the mean dose was less than 10–15 Gy [23]. Miah et al*.* pointed out that whether the mean dose of PGs is greater than 26 Gy can be used to predict xerostomia after RT [26]. Characteristics extracted from DVH, including V15–V45 and D10-D90, were used to predict xerostomia after RT with a good performance [13, 27, 28]. Gabry et al*.* defined the change of mean dose along the coordinate axis as the dose gradient, and applied machine learning technologies to build a superior xerostomia prediction model [29]. Through statistical analysis, it was found that some clinical features are significantly different between patients with xerostomia and normal patients, including age, sex, tumor site, chemotherapy [13, 25].

In above studies, the end point of xerostomia was estimated by grading systems which depends on observer or patient self-evaluation, e.g., EORTC QLQC-30 and H&N35 questionnaire, RTOG criteria. EORTC QLQC-30 and H&N35 questionnaire contains dozens of questions, and uses a 4-point Likert scale to describe the condition as ‘none’, ‘a bit’, ‘quite a bit’, and ‘a lot’ [30]. According to the RTOG criteria, the severity of xerostomia is graded to 5 levels from G0 to G4 [31]. However, the results of the above grading system are qualitative and subjective. Besides, it is not straightforward to compare grades from different systems, because the consistency between them may be low [32]. Additionally, it is suggested that EORTC/RTOG is prone to misinterpretation and omission errors, which underestimates the severity of xerostomia [23, 33, 34]. Thus, we believed that it is more objective and accurate to use saliva amount (SA) reduction to quantify xerostomia. In this study, SA reduction was defined as the stimulated SA difference between 0^th^ fraction and 30^th^ fraction. Moreover, acquiring important features predicting SA reduction is conducive to early intervention of xerostomia after RT.

In previous studies, the evaluation of xerostomia depended on subjective grading systems, rather than the accurate SA reduction. In this study, we used radiomics, DVH criteria and clinical features to establish prediction models for SA reduction. To our knowledge, this is the first study to add DVH criteria and clinical features into radiomics model for predicting SA reduction.

## Materials and method

### Materials

In this study, CT image, DVH, clinical data, and SA_0f-30f_ reduction were collected from 52 NPC patients (24–80 years, median 48 years) who received IMRT in the Sun Yat-sen University Cancer Center (SYSUCC). All patients received radical dosage with a prescription dose of 68.1 Gy in 30 fractions. CT images of all patients at the 0^th^ fraction (planning CT, 0f) and the 10^th^ fraction (10f) during RT were acquired through the CT simulator. PGs contour were delineated on each CT by a radiation oncologist and independently verified by another radiation oncologist. The clinical features included gender, age, tumor stage, BMI, hematological test, blood pressure, etc. Stimulate saliva collection lasted for 5 min at 0^th^ fraction and 30^th^ fraction during RT. Among the 52 patients, 50 patients had reduced SA reduction (maximum SA reduction: 26.2 ml, minimum SA Reduction: 0.1 ml, standard deviation: 6.02), one patient had no change in SA and another patient had increased SA by 0.4 ml. The patients with stages I–IVB, were randomly chosen from the control group in the NPC clinical trial, which was registered on the clinicaltrials.gov (ID: NCT01762514). The study was approved by the institutional review board and the ethical review office from the institution, and the data was submitted to the public scientific research data storage platform (www.researchdata.org.cn). The approval number is RDDB2018000256.

In this study, all CT images were acquired through the CT simulator (Brilliance™ CT, Philips, The Netherlands). The detailed parameters for these protocols were given as following: voltage 120 kVp, exposure 300 mAs, slice thickness 3 mm, increment 3 mm, collimation 16 mm × 0.75 mm, display FOV 600 mm, scan FOV 600 mm, reconstruction filter type UB/B, and pitch 0.567. The DICOM matrix size is 512 × 512 × 113, and the voxel size is 0.9765625 mm × 0.9765625 mm × 3.0000000 mm.

### Feature extraction

The radiomics module of 3D Slicer was used to extract the radiomics features of CT. It had an interface to the PyRadiomics which was an open-source package in python for extracting radiomics features from medical images. With 3D Slicer, we obtained the results of the CT image after the wavelet transform (all combinations of high or low pass filters in each three-dimensional space). Using the radiomics module, we extracted texture features from CT images, including First Order, Shape, Gray Level Co-occurrence Matrix (GLCM), Gray Level Run Length Matrix (GLRLM), Gray Level Size Zone Matrix (GLSZM), Neighbouring Gray Tone Difference Matrix (NGTDM) and Gray Level Dependence Matrix. In this study, we used version 3.0 of PyRadiomics. The initial setting parameters of PyRadiomics are as follows: Spatial Resampling = None, Intensity Rescaling = None, Intensity Discretization: binwidth = 25. And the set wavelet type was haar. All other parameters are default. A total of 3404 dimensions of radiomics features were extracted from all results from 0 and 10f CT and CT wavelet transforms. DVH criteria and clinical features are depicted in Table 1. In Table 1, PGs_V *n* means the volume proportion of the PGs received by *n* Gy. PGs_Dmax, PGs_Dmean and PGs_Dmin are the maximum, mean and minimum values of the dose of PGs respectively. Take PGs_V45 as an example, PGs_V45 represents the left and right PGs, and the volume receiving 45 Gy dose accounts for the proportion of the total volume of the PGs. PGs_Dmax reflects the maximum dose to the whole PGs.

**Table 1:** Statistics of DVH criteria and clinical characteristics

Feature	Mean (Std)	Amount (%)
Sex		
Male	–	38 (73.08)
Female	–	14 (26.92)
Age	48.29 (± 13.46)	–
T stage		
T1	–	4 (8.69)
T2	–	14 (26.92)
T3	–	25 (48.08)
T4	–	9 (17.31)
N stage		
N0	–	10 (19.23)
N1	–	23 (44.23)
N2	–	17 (32.69)
N3	–	2 (3.85)
Clinical stage		
I	–	2 (3.85)
II	–	9 (17.31)
III	–	30 (57.69)
IVa	–	7 (13.46)
IVb		4 (7.69)
Chemotherapy treatment		
1	–	1(1.92)
2	–	45 (89.46)
3	–	6(11.54)
BMI	23.87 (± 2.85)	–
WBC (109 / L)	3.24 (± 0.76)	–
Hb (g / L)	130.26 (± 15.12)	–
PLT (109 / L)	165.50 (± 49.96)	–
Blood calcium (mmol/L)	2.14 (± 0.12)	–
Systolic pressure (mm Hg)	111.40 (± 14.01)	–
Diastolic pressure (mm Hg)	69.14 (± 9.01)	–
PGs_V10 (%)	99.88 (± 0.47)	–
PGs_V15 (%)	98.54 (± 2.19)	–
PGs_V20 (%)	95.74 (± 4.77)	–
PGs_V30 (%)	81.36 (± 8.11)	–
PGs_V40 (%)	66.88 (± 7.23)	–
PGs_V45 (%)	62.94 (± 6.79)	–
PGs_Dmax (cGy)	7053.86 (± 222.01)	–
PGs_Dmean (cGy)	4709.06 (± 531.54)	–
PGs_Dmin (cGy)	1551.53 (± 491.51)	–

PGs_V *n* means the volume proportion of the PG received by *n* Gy. PGs_Dmax, PGs_Dmean and PGs_Dmin are the maximum, mean and minimum values of the dose of PG respectively

*WBC* white blood cell, *Hb* hemoglobin, *PLT* platelet

### Feature selection

In this study, there were high dimensional features. If all features were used for modeling, the generalization of the model will be limited. Thus, feature selection was essential for developing the generalization. Besides, in this study, there were great differences in the order of magnitude of radiomics, DVH criteria and clinical features (radiomics: 3404, DVH criteria: 9, clinical: 15). If the 3 groups of features are selected together, radiomic features will obscure the importance of DVH criteria and clinical features. Therefore, we referred to the practice of Yu et al. [35], and carried out feature selection on these 3 groups of features respectively. In previous studies, the most common feature pre-selection was to retain the features with the highest correlation with the predicted target, in the interval where Pearson correlation was more than 0.8 [7, 15, 17]. Since the Pearson correlation method cannot provide more information between the features and the predicted targets, eXtreme Gradient Boosting (XGBoost) + SHapley Additive exPlanations (SHAP) was chosen to select the features and explore the features influence on the predicted target, even if that feature was not used for modeling finally.

**Fig. 1:**
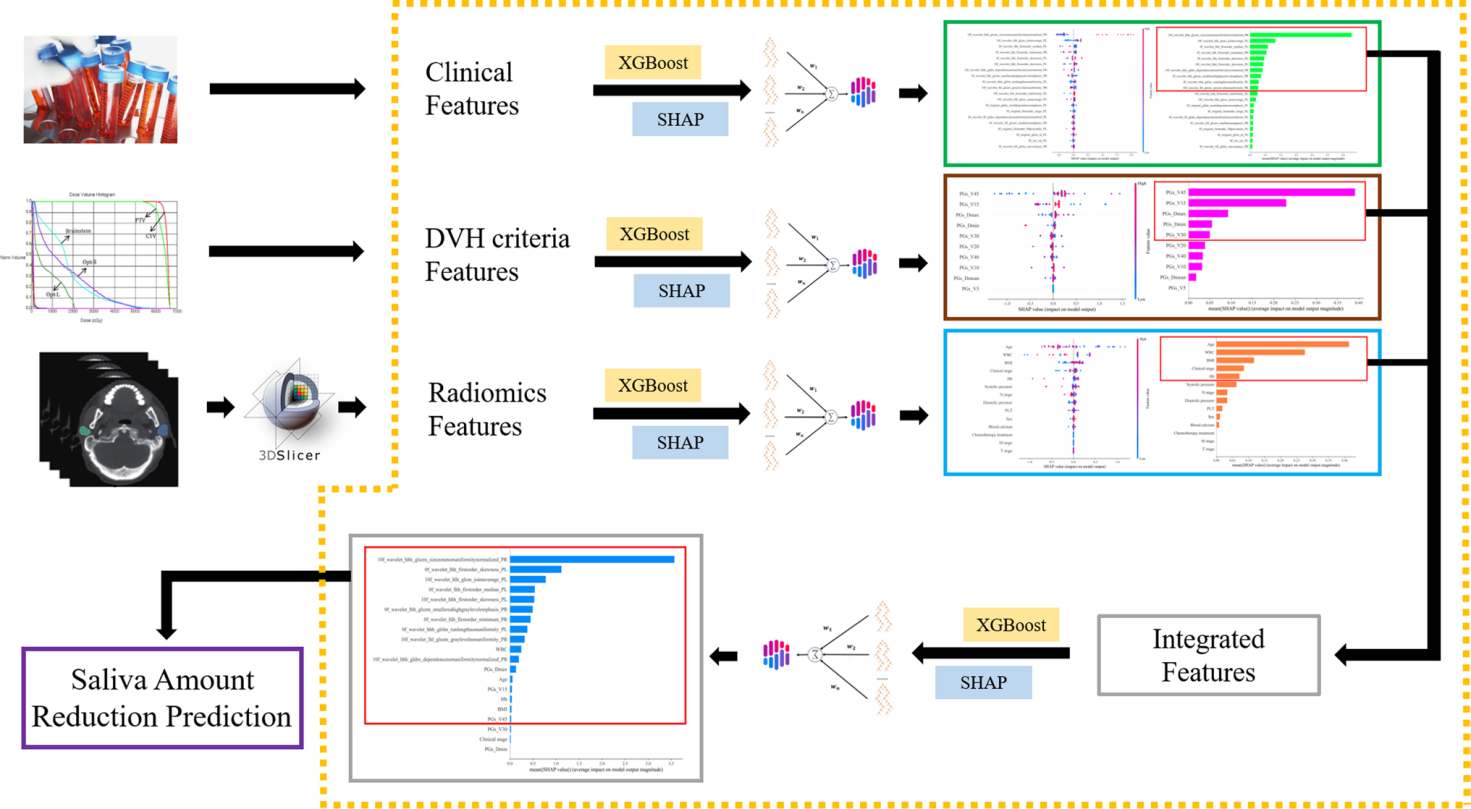
Feature Selection

The procedures of XGBoost + SHAP are as follows. First, the prediction models of SA reduction were established by applying XGBoost to radiomics, DVH criteria and clinical features, respectively. Second, for each of the three models, SHAP analysis was used to obtain the weights of feature importance within the groups. Third, with the guidance of radiation oncologists, the top-ranked features of the three groups were selected. The principle of selection was to include most of the feature importance with fewer features. Fourth, the selected radiomics, DVH criteria and clinical features composed integrated features. For integrated features, another model was established by XGBoost, which was analyzed using SHAP then. The purpose was to obtain the feature importance of integrated features and further select the features. The principle of selection was consistent with step 3. The flowchart was shown in the yellow dotted box in Fig. 1.

### XGBoost

Similar to gradient boosting decision tree (GBDT), XGBoost continuously reduces the bias of the additive model by fitting previous base model prediction errors with the new base model. Compared with the traditional GBDT, XGBoost takes the loss function to a second-order Taylor expansion instead of a first-order one, while adding regularization term to the loss function [36]. As a result, XGBoost has better predictive and generalization capabilities. Bianchi et al*.* found that XGBoost model can diagnose earlier osteoarthritis of the temporomandibular joint, based on biomarkers [37]. The loss function is shown in Eq. (1).

1 $${L}^{(t)}=\sum_{i=1}^{n}[{g}_{i}{f}_{t}\left({x}_{i}\right)+\frac{1}{2}{h}_{i}{{f}_{t}}^{2}\left({x}_{i}\right)]+\gamma T+\frac{1}{2}\sum_{j=1}^{T}{{w}_{j}}^{2}$$

wherein, ${g}_{i}={\partial }_{{\widehat{y}}^{(t-1)}}l({y}_{i},{\widehat{y}}^{(t-1)})$, ${h}_{i}={\partial }_{{\widehat{y}}^{(t-1)}}^{2}l({y}_{i},{\widehat{y}}^{(t-1)})$, are respectively the first and second partial derivatives of the loss function. $\gamma T+\frac{1}{2}\sum_{j=1}^{T}{{w}_{j}}^{2}$ is the regularization item.

### SHAP analysis

Inspired by cooperative game theory, SHAP [38] analysis constructs an additive explanatory model, which obtain the independent importance of each feature. Considering the influence and synergy between variables, it shows the average contribution margin of each feature, which effectively avoid multicollinearity. SHAP can not only obtain the overall importance of a feature, but also be used to reflect the influence of a feature in different samples. For each prediction sample, SHAP analysis produces a predicted value. It is the sum of the values assigned to each feature, as shown in Eq. (2).

2 $${y}_{i}={y}_{base}+f\left({x}_{i,1}\right)+f\left({x}_{i,2}\right)+\dots +f\left({x}_{i,k}\right)$$

wherein,${y}_{i}$ is the SHAP value of the model for the $i$ th sample, and ${y}_{base}$ is the mean SHAP value of all samples. ${x}_{i,j}$ means the $j$ th feature of the $i$ th sample.$f\left({x}_{i,j}\right)$, is the SHAP value of ${x}_{i,j}$, reflecting the contribution of ${x}_{i,j}$ to the final SHAP value ${y}_{i}$. When $f\left({x}_{i,j}\right)$>0, it indicates that the feature improves the final SHAP value ${y}_{i}$, i.e., the positive effect; When $f\left({x}_{i,j}\right)$<0, it indicates that this feature reduces the final SHAP value ${y}_{i}$, i.e., the negative effect.

### Prediction model

After feature selection, SA reduction model was established by a variety of machine learning technologies to find the most accurate model. Besides, comparing the performance differences of models established by different algorithms and different feature groups, the influence of DVH criteria and clinical features on predicting SA reduction was explored.

### Ridge regression

Ridge adds L2-norm [39] to linear regression loss function, which is beneficial to alleviate the problem of multicollinearity and overfitting. Using radiomics features, Wei et al*.* used Ridge to predict the prognosis of skull base chordoma [40]. The loss function of ridge is shown in Eq. (3).

3 $$J=\frac{1}{n}\sum_{i=1}^{n}{(f\left({x}_{i}\right)-{y}_{i})}^{2}+\lambda {||\upomega ||}_{2}^{2}$$

wherein, $\lambda {||\upomega ||}_{2}^{2}$ is the L2-norm. As $\lambda$ increase, the generalization capability of the model is strengthened.

### Support vector regression (SVR)

SVR seeks a regression hyperplane that minimizes the distance of all the data from that hyperplane [41]. It is suitable for cases with linearly indivisible samples and high characteristic dimensions.

### Decision tree

Decision Tree constructs binary trees to make decisions [42]. It is adept at dealing with the nonlinear relationship and outliers in the data. Sakai et al*.* used decision tree to detect multi-leaf collimator (MLC) modeling errors with the use of radiomic features [43].

### Random forest

Random Forest is an algorithm integrated by bagging of multiple decision trees [44]. There is no correlation between each decision tree, and the final output of the model is jointly determined by every decision tree. Since the feature subset of each decision tree is randomly selected, it has good robustness and can maintain accuracy even if data are missing. Homayounieh et al*.* used random forest to differentiate diffuse liver diseases on non-contrast CT [45].

### Adaboost

AdaBoost is a boosting ensemble algorithm with two characteristics [46]. First, samples predicted wrong by previous base model are given high weight to increase the attention of the next base model.

Second, the base models with high accuracy are given higher weight, and the final output is the weighted average output of multiple base models. As a result, AdaBoost has an outstanding prediction performance. Thongkam et al*.* predicted breast cancer survivability by AdaBoost [47].

## Result

**Fig. 2:**
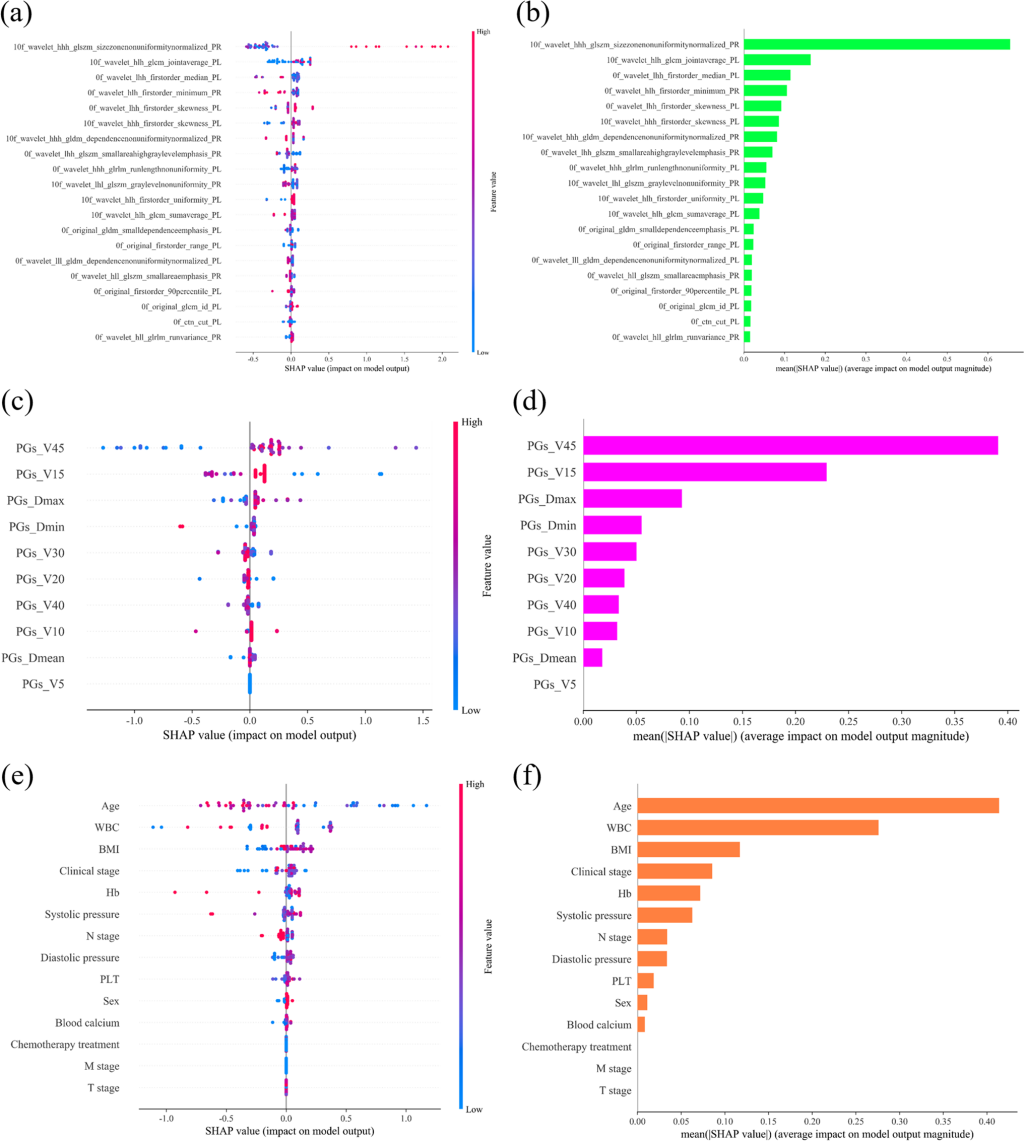
SHAP Analysis of Radiomics, DVH criteria, and Clinical Features. **a** Distribution of SHAP values for radiomic features; **b** Ranking of SHAP values for radiomic features; **c** Distribution of SHAP values for DVH criteria features; **d** Ranking of SHAP values for DVH criteria features; **e** Distribution of SHAP values for clinical features; **f** Ranking of SHAP values for clinical features; **a**, **b**, ‘0f_’, ‘10f_’respectively mean the radiomics features extracted from 0^th^ fraction CT and 10^th^ fraction CT. ‘PL’ and ‘PR’ respectively mean the left parotid gland and right parotid gland. *WBC* white blood cell, *Hb* hemoglobin, *PLT* platelet

In this study, the definition of SA reduction is shown in Eq. (4). First, radiomics, DVH criteria and clinical features were modeled by XGBoost, and the three models were analyzed by SHAP. The result was shown in Fig. 2.

4 $${SA}_{0f-30f} recution={SA}_{0f}-{SA}_{30f}$$

In Fig. 2. (c), for PGs_V45, the blue dots are clustered to the left of the longitudinal axis. Blue indicates that the sample has a small value of PGs_V45. The points to the left of the axis all have SHAP values less than zero, indicating that they have the negative effect on the prediction. Therefore, it can be concluded that PGs_V45↓, (SA0f–SA30f) ↓, SA30f ↑, severity of xerostomia ↓. Meanwhile, a lot of red dots are concentrated on the right side of the vertical axis. The red color indicates that the value of PGs_V45 for this sample is large. The dots to the right of the axis, which have SHAP values greater than zero, have a positive effect on the prediction. It is indicating that PGs_V45 ↑, (SA0f–SA30f) ↑, SA30f ↓, severity of xerostomia ↑. Generally, PGs_V45 is positively correlated with the severity of xerostomia. Similarly, in Fig. 2e, it is suggested age was negatively correlated with the severity of xerostomia.

Figure 2b, d, f show the weights of important features in radiomics, DVH criteria, and clinical features, respectively. With the participation of radiation oncologists, the top 10 radiomics, the top 5 DVH criteria, and the top 5 clinical features were selected for the second feature selection. The reason is that fewer features can effectively avoid overfitting. We expect to retain no more than 10 radiomic features to reduce the risk of overfitting. In addition, due to the close number of DVH criteria and clinical features, we set them to equal weight. Meanwhile, in order to investigate the influence of adding DVH criteria and clinical features based on radiomic features, we set the total number of DVH criteria and clinical features consistent with radiomic features. Therefore, from Fig. 2b, d, f, the top 10 radiomics features, the top 5 DVH criteria features and the top 5 clinical features respectively contain most of the importance of the features, which reflects most of the information in all features.

**Fig. 3:**
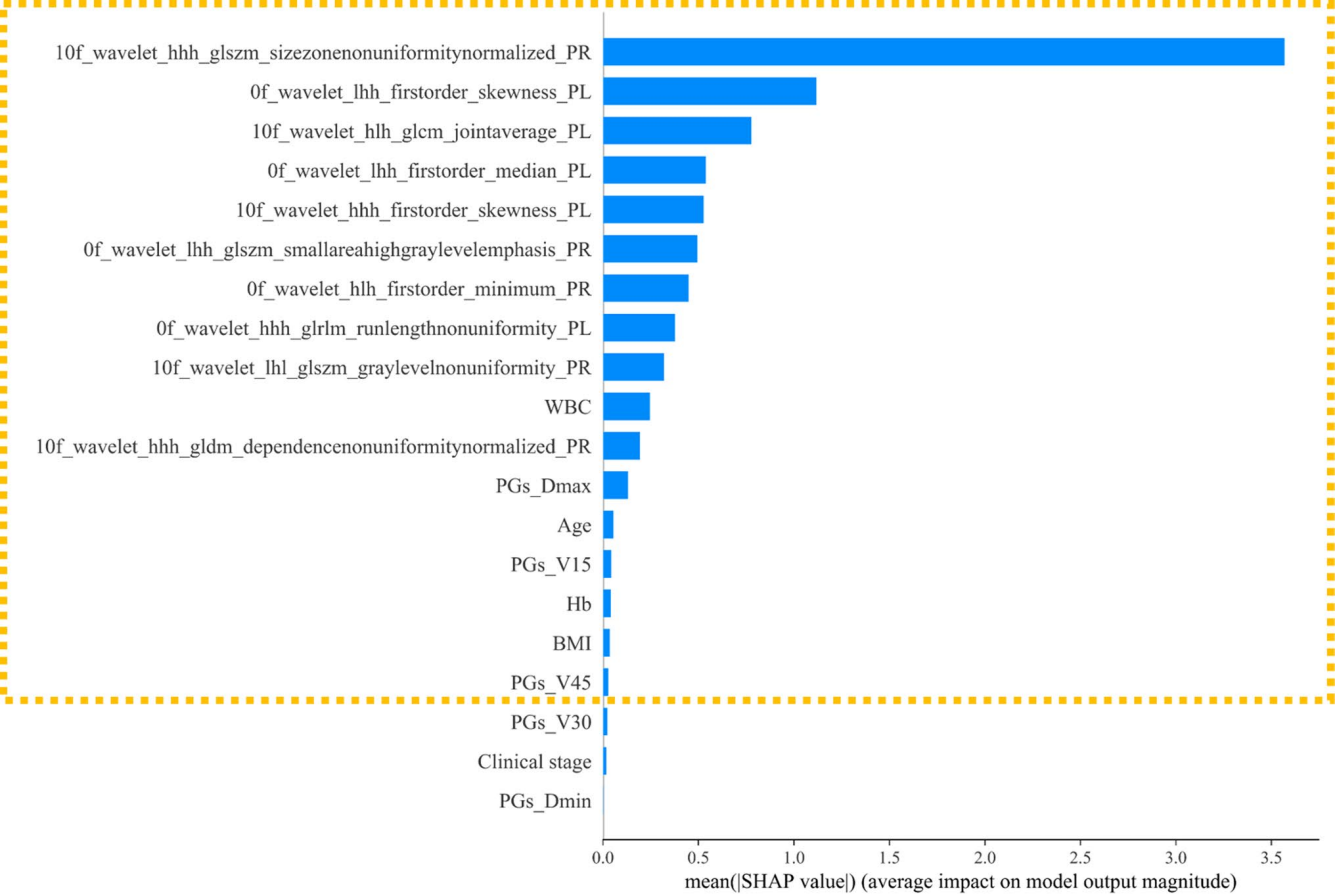
SHAP analysis of integrated features

**Fig. 4:**
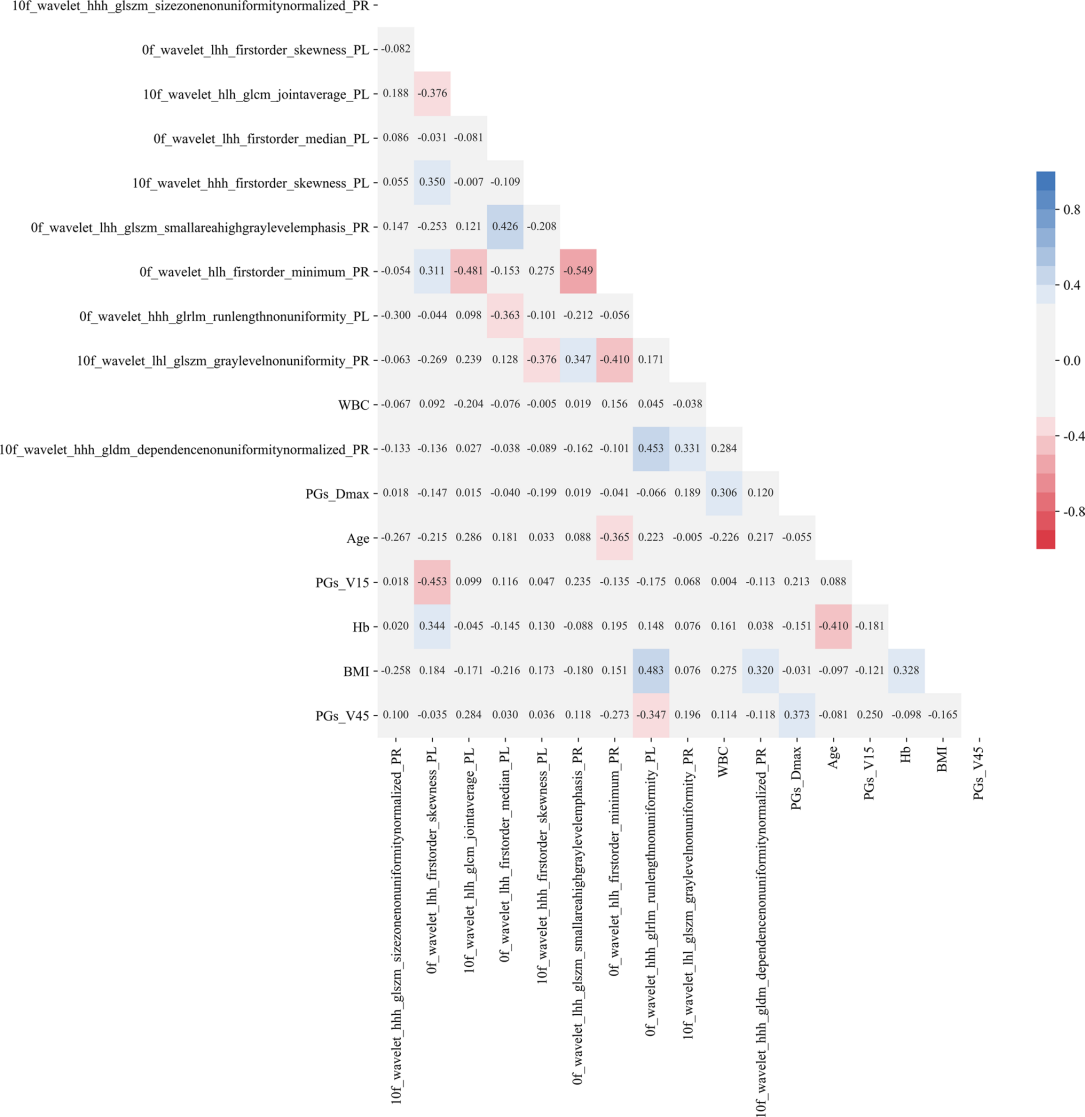
Pearson correlation heat map of top 17 integrated features

The above 20 features were selected again by XGBoost + SHAP, and the rank of feature importance was shown in Fig. 3. The top 17 features (shown in the yellow dotted box in Fig. 3) were selected while the top 10 radiomics features were included in them. We calculated the Pearson correlation of these 17 features, and the results are shown in Fig. 4.

To avoid multicollinearity, the most commonly method was to retain the feature with the highest correlation to the predicted target among the features with Pearson correlation greater than 0.8. In Fig. 4, it is observed that the Pearson correlations between any features selected using XGBoost + SHAP are less than 0.8. Among the 17 features, the Pearson correlation between “0f_wavelet_lhh_glszm_small-areahighgraylevelemphasis_PR” and “0f_wavelet_hlh_firstorder_minimum_PR” has the largest Pearson correlation of 0.549.

It is concluded that XGBoost + SHAP can effectively avoid feature multicollinearity. Then, we used Ridge, SVR, Decision Tree, Random Forest, AdaBoost, and XGBoost to establish the prediction model on SA reduction with these 17 features. Additionally, models were constructed using the top 10 and 17 radiomics features to compare the effect of DVH criteria and clinical features. The 17 integrated (10 radiomics, 4 clinical, 3 DVH criteria), top 17 radiomics, and top 10 radiomics features are shown in Table 2.

**Table 2:** The 17 integrated, top 17 radiomics and top 10 radiomics features

Feature name	Top 17 integrated	Top 17 radiomics	Top 10 radiomics
10f_wavelet_hhh_glszm_sizezonenonuniformitynormalized_PR	√	√	√
0f_wavelet_lhh_firstorder_skewness_PL	√	√	√
10f_wavelet_hlh_glcm_jointaverage_PL	√	√	√
0f_wavelet_lhh_firstorder_median_PL	√	√	√
10f_wavelet_hhh_firstorder_skewness_PL	√	√	√
0f_wavelet_lhh_glszm_smallareahighgraylevelemphasis_PR	√	√	√
0f_wavelet_hlh_firstorder_minimum_PR	√	√	√
0f_wavelet_hhh_glrlm_runlengthnonuniformity_PL	√	√	√
10f_wavelet_lhl_glszm_graylevelnonuniformity_PR	√	√	√
10f_wavelet_hhh_gldm_dependencenonuniformitynormalized_PR	√	√	√
WBC	√		
PGs_Dmax	√		
Age	√		
PGs_V15	√		
Hb	√		
BMI	√		
PGs_V45			
10f_wavelet_hlh_firstorder_uniformity_PL		√	
10f_wavelet_hlh_glcm_sumaverage_PL		√	
0f_original_gldm_smalldependenceemphasis_PL		√	
0f_original_firstorder_range_PL		√	
0f_wavelet_lll_gldm_dependencenonuniformitynormalized_PL		√	
0f_wavelet_hll_glszm_smallareaemphasis_PR		√	

‘PL’ and ‘PR’ respectively mean the left parotid gland and right parotid gland

**Fig. 5:**
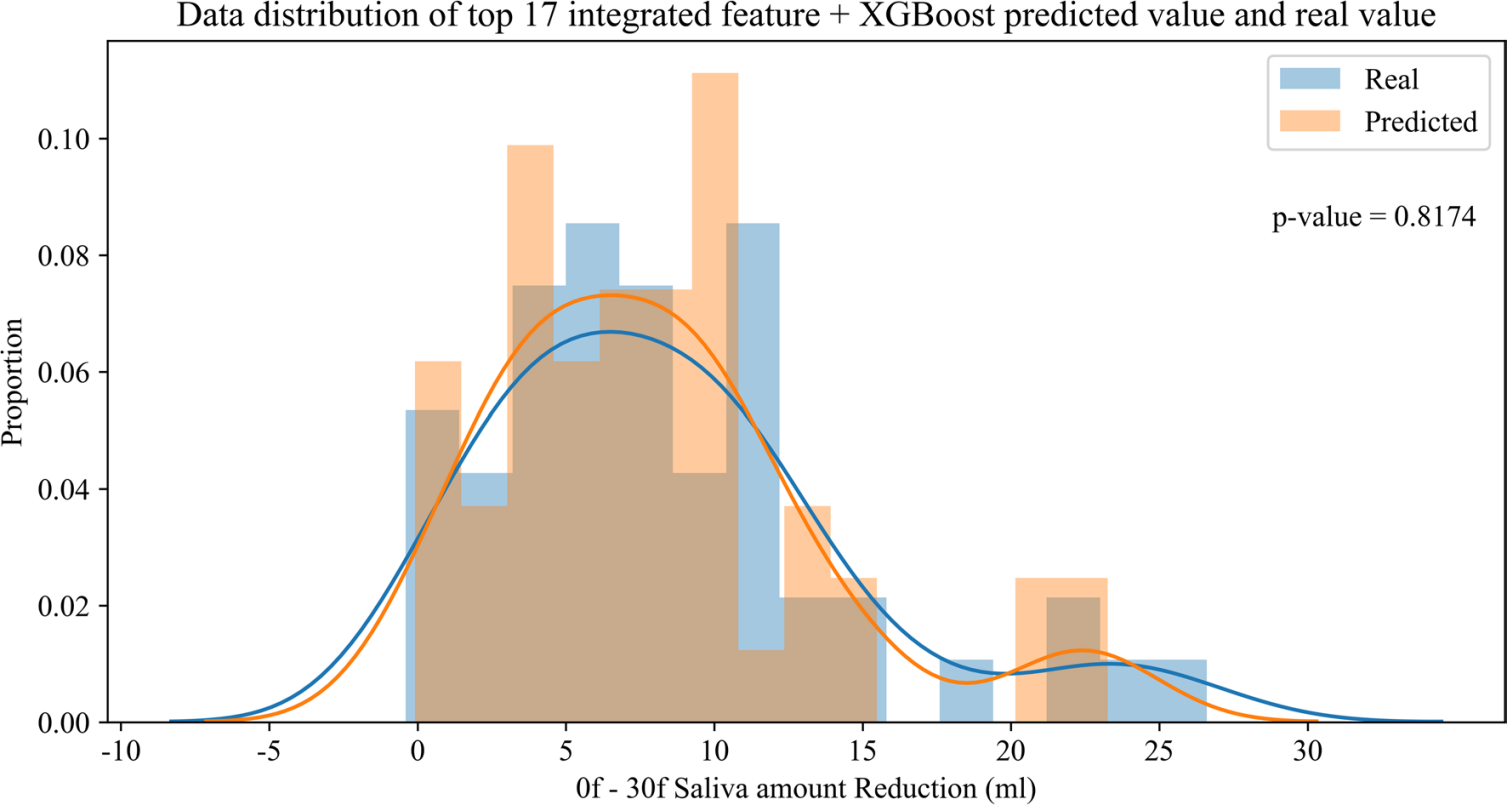
Top 17 integrated features_XGBoost predicts the SA reduction

Because of the small sample size in this study, we used the leave-one-out method to validate the predictive performance of the model. Specifically, 51 patients were used for training, and the remaining one was tested, which repeated 52 times. MSE and R^2^ were used as the evaluation metrics. The performances are shown in Table 3. Based on the top 17 integrated features, the distribution of predicted values applying XGBoost versus the distribution of real values is shown in Fig. 5.

**Table 3:** The performance of predicting SA reduction

Algorithms	Top 17 integrated features		Top 17 radiomics features		Top 10 radiomics features	
	MSE	R^2^	MSE	R^2^	MSE	R^2^
XGBoost	0.6994	0.9815	0.7376	0.9805	0.7519	0.9801
AdaBoost	1.4610	0.9614	1.6538	0.9563	1.8341	0.9515
Random Forest	2.4580	0.9350	2.7202	0.9281	3.0747	0.9187
Decision Tree	2.5643	0.9322	3.3712	0.9109	3.0713	0.9188
SVR	11.3157	0.7009	15.6761	0.5857	17.0220	0.5501
Ridge	17.9751	0.5249	18.9739	0.4985	22.3401	0.4095

Top 17 integrated features, including top 10 radiomics, 4 clinical and 3 DVH criteria features

The experimental results showed that the prediction model of SA reduction based on XGBoost using top 17 integrated features (including top 10 radiomics features, 4 clinical, and 3 DVH criteria features) had the highest accuracy. In addition, we observed that applying the same algorithm, top 17 integrated features performed better than the other two feature groups.

## Discussion

Most of studies used Pearson correlation method to pre-select features, but it ignored the information carried by the removed features. However, with XGBoost + SHAP, researchers can explore the impact of each feature, whether or not these features are ultimately used for modeling. In this study, PGs_V30, clinical stage, PGs_Dmin were ranked low in feature importance and were not used for modeling. But through XGBoost + SHAP, from Figs. 2c, e, we can explore the influence of these factors on SA reduction after RT, which enables researchers to obtain more comprehensive information.

Numerous studies showed that radiomics features can be used to predict xerostomia after RT with an accurate performance [7, 8, 10, 17, 34]. Similarly, in this study, XGBoost, AdaBoost, Random Forest, Decision Tree, SVR, and Ridge were applied to predict SA_0f-30f_ reduction based on only radiomics features. All R^2^ from the models established by XGBoost, AdaBoost, Random Forest and Decision Tree were greater than 0.90, indicating that the models have high accuracy. It further supports that CT-based radiomics features are important in predicting the risk of xerostomia [19]. Previous studies showed that DVH criteria and clinical features also played an important role in predicting xerostomia after RT. To our knowledge, this is the first study to use radiomics, DVH criteria and clinical features to predict SA reduction. Adding DVH criteria and clinical features to radiomics features, the performance of all machine learning models was improved, which was similar to the findings of Sheikh et al. [48]. The results of applying different machine learning techniques with the same number of features showed that using integrated features (radiomics + DVH criteria + clinical) can improve the accuracy of the model than using only radiomics features. All in all, we believe that using radiomics, DVH criteria and clinical features to predict SA reduction after RT has huge potential value because this approach not only improves prediction performance but also increases the interpretability of the model. In this study, we used radiomics, DVH criteria and clinical features to predict SA reduction after RT. The best model is XGBoost with an MSE of 0.6994 and R^2^ of 0.9815.

In this study, we observed that PGs_Dmax, PGs_V15, and PGs_V45 were positively correlated with the severity of xerostomia. It was consistent with a large number of studies showing that the lower the mean dose of PGs, the lower the degree of xerostomia [22, 25–27]. There were fewer studies using PGs_Dmax, PGs_V15, and PGs_V45, but these characteristics reflect the dose to which the PGs are exposed. It was generally accepted that the high amount of radiation to which the PGs is exposed, increases the risk of radiation damage to PGs.

In this study, age also played an important role in predicting SA reduction after RT. It was widely accepted that age is an important prediction factor of xerostomia. However, there is still controversy regarding the relationship between age and xerostomia. Some studies suggested that age is negatively associated with xerostomia significantly [12, 25]. And some studies suggested that patients with large age have an increased risk of xerostomia due to the use of certain medications [49]. In this study, from Fig. 2c, it is showed that with the decrease of age, the severity of xerostomia increased. We believed that it may be related to the differences between the study samples. Meanwhile, we found that Hb had an important influence on the prediction of SA reduction. Currently, there is a lack of studies on the relationship between Hb level and xerostomia after RT in NPC patients. It is reported that an increase Hb concentration enhances the radiosensitivity of normal tissue such as skin and mucosa [50], which may increase PGs damage, leading to obvious SA reduction. Additionally, in this study, BMI was significant for predicting SA reduction after RT. Egestad et al*.* showed that patients with BMI ≥ 25 had more problems with xerostomia after RT than patients with BMI < 25 [51]. Sanguineti et al*.* also found that patients with a BMI > 30 had a significantly higher risk of xerostomia after RT [52]. The conclusions of the above studies are similar to those of this study. From Fig. 2c, it can be observed that BMI has a positive effect on SA reduction. Possibly, obese patients might experience relatively larger changes in anatomy, which might cause the salivary glands to receive higher doses [53].

In particular, our study has some limitations. First, we used radiomics features of PGs to predict SA reduction after RT. It is reported that about 60–70% of the stimulated SA originates from PGs, about 20% from the submandibular gland, and the rest from the other salivary glands [54]. Thus, we will add radiomics features of submandibular gland to the model to explore the role of submandibular gland. Second, this study was based on 52 NPC patients, and the small number of patients limited the generalizability of the models, so the validation on larger data sets will be needed in the future.

## Conclusion

In this study, we used XGBoost + SHAP to feature selection. It can avoid multicollinearity while assisting the researcher to understand the impact of all features on the prediction target. We believed that it can be extended to studies predicting other diseases. Based on the radiomics features, adding DVH criteria and clinical features can effectively improve the accuracy of the model in predicting SA reduction. With the same number of features, using integrated features (radiomics + DVH criteria + clinical) can achieve better prediction performance than using only radiomics features. In this paper, the optimal combination of models for SA reduction is XGBoost + top 17 integrated features. The important DVH criteria and clinical features include WBC, PGs_Dmax, Age, PGs_V15, Hb, BMI and PGs_V45.

## Data Availability

The data that support the findings of this study are available from [The public scientific research data storage platform (www.researchdata.org.cn).], and the approval number is RDDB2018000256. But restrictions apply to the availability of these data, which were used under license for the current study, and so are not publicly available. Data are however available from the authors upon reasonable request and with permission of [The public scientific research data storage platform].
